# Fleroff goes digital: georeferenced records from "Flora des Gouvernements Wladimir" (Fleroff, 1902)

**DOI:** 10.3897/BDJ.9.e75299

**Published:** 2021-10-20

**Authors:** Alexey P. Seregin, Yurii M. Basov

**Affiliations:** 1 M.V. Lomonosov Moscow State University, Moscow, Russia M.V. Lomonosov Moscow State University Moscow Russia; 2 Unaffiliated, Tyumen, Russia Unaffiliated Tyumen Russia

## Abstract

**Background:**

Global Biodiversity Information Facility (GBIF) has uneven data coverage across taxonomic, spatial and temporal dimensions. Temporal imbalances in the data coverage are particularly dramatic. Thus, 188.3M GBIF records were made in 2020, more than the whole lot of the currently available pre-1986 electronic data. This underscores the importance of reliable and precise biodiversity spatial data collected in early times. Biological collections certainly play a key role in our knowledge of biodiversity in the past. However, digitisation of historical literature is underway, being a modern trend in biodiversity data mining. The grid dataset for the flora of Vladimir Oblast, Russia, includes many historical records borrowed from the "Flora des Gouvernements Wladimir" by Alexander F. Fleroff (also known as Flerov or Flerow). Intensive study of Fleroff's collections and field surveys exactly in the same localities where he worked, showed that the quality of his data is superb. Species lists collected across hundreds of localities form a unique source of reliable information on the floristic diversity of Vladimir Oblast and adjacent areas for the period from 1894 to 1901. Since the grid dataset holds generalised data, we made precise georeferencing of Fleroff's literature records and published them in the form of a GBIF-mediated dataset.

**New information:**

A dataset, based on "Flora des Gouvernements Wladimir. I. Pflanzengeographische Beschreibung des Gouvernements Wladimir" by Fleroff (1902), includes 8,889 records of 654 taxa (mainly species) from 366 localities. The majority of records originate from Vladimir Oblast (4,611 records of 534 taxa from 195 localities) and Yaroslavl Oblast (2,013 records of 409 taxa from 66 localities), but also from Nizhny Novgorod Oblast (942 records), Ivanovo Oblast (667 records) and Moscow Oblast (656 records). The leading second-level administrative units by the number of records are Pereslavsky District (2,013 records), Aleksandrovsky District (1,318 records) and Sergievo-Posadsky District (599 records). Georeferencing was carried out, based on the expert knowledge of the area, analysis of modern satellite images and old topographic maps. For 2,460 records, the georeferencing accuracy is 1,000 m or less (28%), whereas for 6,070 records it is 2,000 m or less (68%). The mean accuracy of records of the entire dataset is 2,447 m. That accuracy is unattainable for most herbarium collections of the late 19^th^ century. Some localities of rare plants discovered by Fleroff and included into the dataset were completely lost in the 20^th^ century due to either peat mining or development of urban areas.

## Introduction

**Figure 1.**
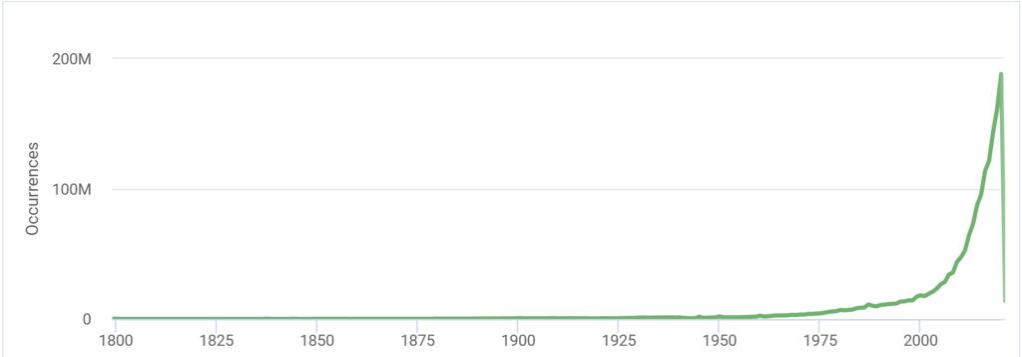
Distribution of GBIF-mediated records from 1800 to 2021 by years showing the disproportion in temporal data coverage across GBIF (source: https://www.gbif.org/, as of 05 September 2021).

GBIF has uneven data coverage across taxonomic, spatial and temporal dimensions. Temporal imbalances in the data coverage are particularly dramatic (Fig. 1). They resulted from the intensification of the biodiversity documentation in the 20^th^ century and the explosive growth of crowdsourcing platforms in the 21^st^ century. Thus, in GBIF, there are 188,334,269 records made in 2020, more than the whole lot of the currently available pre-1986 electronic data (186,905,290 records). This underscores the importance of reliable and precise biodiversity spatial data collected in the 19^th^ century and earlier.

Biological collections certainly play a key role in our knowledge of biodiversity in the past. In GBIF, 8.23 M out of 10.54 M pre-1900 records are based upon museum specimens. Nonetheless, digitisation of literature is underway. Direct on-purpose digitisation and transcription into the form of GBIF-mediated data of published sources is a modern trend in biodiversity data mining. In particular, numerous datasets from Plazi.org platform (https://www.gbif.org/publisher/7ce8aef0-9e92-11dc-8738-b8a03c50a862) contributed 480,751 occurrences from taxonomic treatment articles.

In the Russian segment of GBIF, digitised points from the printed atlases are the largest datasets based upon literature sources. For instance, dot maps from the "Flora of Siberia" (Artemov and Egorova 2021), "Flora of Murmansk Region" (Kozhin et al. 2020, Kozhin et al. 2021) and atlas of the "Endemic Alpine Plants of Northern Asia" (Brianskaia et al. 2021a, Brianskaia et al. 2021b) were completely transcribed into the electronic datasets.

**Vladimir Oblast in GBIF.** Vladimir Oblast (29,084 km^2^) is the first-level administrative unit of the Russian Federation situated east of Moscow. This is a region with a high density of GBIF-mediated data on floristic diversity. To date, 188,790 records of tracheophytes originated from Vladimir Oblast out of 3,437,051 records available for the flora of Russia. Average data density on vascular plants from this area is 6.49 records per 1 km^2^. The most extensive datasets are:

1. Flora of Vladimir Oblast, Russia: an updated grid dataset (1867–2020) (Seregin 2021a, Seregin 2021b);
2. iNaturalist Research-grade Observations (Ueda 2021, see also Seregin et al. 2020);
3. A grid-based database on vascular plant distribution in the Meshchera National Park, Vladimir Oblast, Russia (Seregin 2014a);
4. Moscow University Herbarium (MW) (Seregin 2021c).

The largest grid dataset with ca. 130 K records (Seregin 2021a, Seregin 2021b) served earlier as the basis for the standard flora of the region (Seregin 2012) with many historical records borrowed from the old standard flora by Fleroff (1902). The records obtained from Fleroff (1902) contributed to that dataset being georeferenced to grid-square centroids with accuracy of records equalling 7,000 m. Being merged with other data in generalised form, Fleroff's records cannot be separated from the main bulk of information. Only with the present dataset are these historical data traceable and recognisable. In addition, Seregin (2012) did not process some records by Fleroff (1902) at all, since certain areas of the former Vladimir Governorate were excluded from the modern Vladimir Oblast.

**Figure 2.**
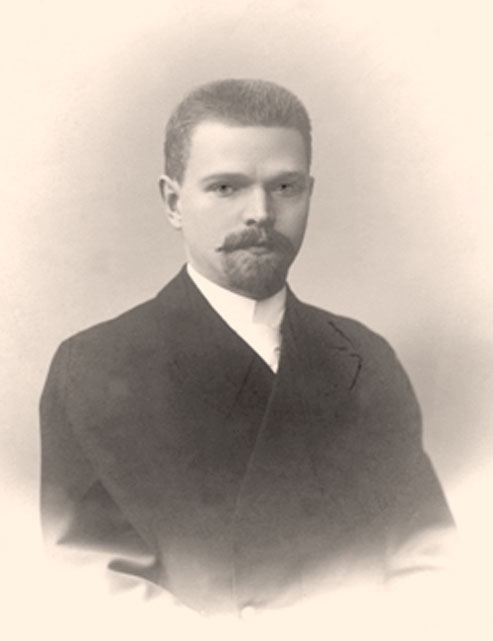
Alexander F. Fleroff while working in Vladimir Governorate.

The experience of the author's (A.P. Seregin) work on the grid atlas, his intensive study of Fleroff's herbarium collections and field surveys exactly in the same localities where Alexander F. Fleroff (Fig. 2) worked, showed that quality of his data is superb. Species lists collected across hundreds of localities and published by Fleroff (1902) are forming a unique source of reliable information on the floristic diversity of Vladimir Oblast and adjacent areas for the late 19^th^ century and the very beginning of the 20^th^ century. Since the grid dataset by Seregin (2021b) holds generalised grid data, it is time to return to Fleroff (1902) and make accurate georeferencing of his numerous high-quality records.

**Spelling of the surname.** In modern standards, the Russian surname "Флёров" could be transcribed into English as "Flerov" following the spelling (BSI standard) or "Flyorov" following the pronunciation (GOST 7.79-2000). However, in the past, it was a common practice to use "-off" ending for the Russian surnames like "Sokoloff" (Соколов), "Smirnoff" (Смирнов) etc. In his book, Fleroff (1902) used "Fleroff" on the title pages, therefore this orthographic variant is used here throughout.

However, IPNI suggests another forms as standard ones, like "Flerow" (urn:lsid:ipni.org:authors:2781-1,  https://www.ipni.org/a/2781-1) for tracheophytes and "Flerov" (urn:lsid:ipni.org:authors:20035717-1,  https://www.ipni.org/a/20035717-1) for fungi. These both LSIDs refer to him.

## General description

### Purpose

The purpose of this newly-created dataset (Seregin and Basov 2021) is to deliver to a wider audience in the form of GBIF-mediated data the vast floristic materials collected and published by Fleroff (1902) across various localities of Vladimir Governorate. To make this, we digitised species lists for ca. 500 individual localities/plant communities from the original source and made their georeferencing.

**Figure 3.**
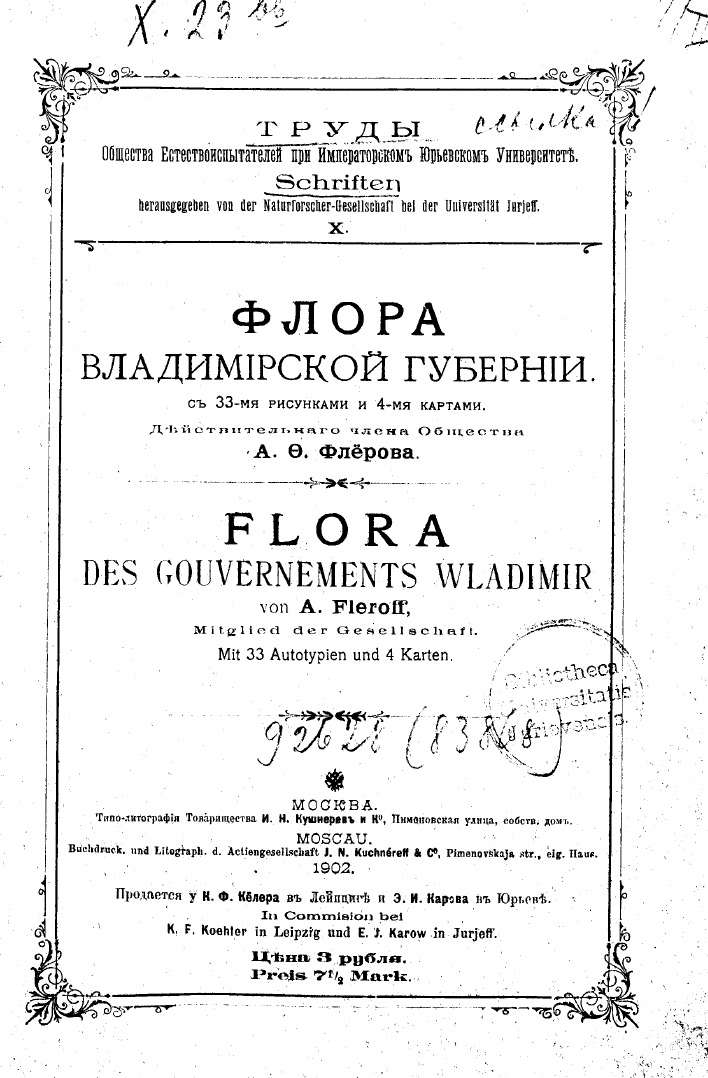
Title page of the original source by Fleroff (1902), a monograph published in Moscow within the "Schriften herausgegeben von der Naturforscher-Gesellschaft bei der Universität Jurieff" series.

**Structure of the original source**: The book "Flora of Vladimir Governorate" by Fleroff (1902) consists of two parts with independent paginations within a single monograph (Fig. 3). This form was used by Bulatkin (1896) for the flora of south-eastern districts of the Governorate and obviously repeated by Fleroff.

The first part is written in two languages, i.e. the main text in Russian (338 pages) with the extended summary in German (18 pages) (Fig. 4a). It is subtitled "Описание растительности Владимирской губернии" ("Description of the vegetation of Vladimir Governorate"), but the German subtitle makes a different accent, i.e. "Pflanzengeographische Beschreibung des Gouvernements Wladimir" ("Description of the plant geography of Vladimir Governorate"). The German abstract is devoted to general questions of plant geography of the area and includes mostly the discussion and conclusions.

From the point of view of a 21^st^ century researcher, the most important fragments of the first part are lists of species in Latin for individual communities with a clear indication of localities (Fig. 5). In fact, these are simple relevés, which were digitised and georeferenced by us. The length of these relevés depends on a variety of reasons. Communities can be species-rich (like floodplain meadows or hardwood forests) or species-poor (like oligotrophic lakes or dry pine forests), a description could be thorough and time-consuming or made in the form of short notes along the route, it could cover a small lake or a large forest. In addition, certain noteworthy species were mentioned by Fleroff in addition to regular species lists.

The second part of the Fleroff's book is a checklist written in Latin on 70 pages and entitled "Flora Gubernii Wladimiriensis. II. Enumeratio plantarum" (Fig. 4b). The checklist is typeset in petite and includes 881 numbered species of the flora of Vladimir Governorate. Each species has a short description in three or four lines (Fig. 6), including:

- number (from 1 to 881);
- accepted Latin name with taxonomic authors;
- occasional synonymy;
- data source ("!!" for Fleroff's own data, "!" for herbarium collections of other researchers and unmarked for published references);
- bibliographic citations with a page reference (Zinger 1885, Bulatkin 1896, Schmalhausen 1895 for all species as well as some occasional extra references for rare species);
- habitat details;
- frequency ("copiosissime", "frequens", "ubique frequens", "rarum apud nos" etc.);
- list of districts for rare species (with references, if necessary);
- indication of localities for the rarest species;
- infraspecific taxa (if any);
- pharmacopoeial name (like "Semen Lycopodii" etc.).

Fleroff intensely revised the nomenclature of the checklist prior to its publication. He made some adjustments and name substitutions according to the recently-published monographs. Therefore, he altered some names widely used in the first part (like *Betonicaofficinalis* L., *Clinopodiumvulgare* L., *Orobusvernus* L. etc.). Later, species entries from the second part of Fleroff (1902) were cited in the nomenclatural paragraphs by Seregin (2012). Since the second part of the original source does not contain additional individual records, we have not georeferenced it for the dataset. The checklist ends with two lists of herbarium collections from Vladimir Governorate, i.e. (1) processed by Zinger (1885) (38 collections, at least 5,700 specimens) and (2) sent to Fleroff (11 collections, at least 1,075 specimens). Zinger's personal herbarium is currently deposited at the Moscow University Herbarium (MW).

In 1902, Fleroff (1902) defended his monograph as a Master's Thesis at the Yuryev University (currently Tartu University) and was awarded a Master's Degree in botany. At the request of Professor N.I. Kuznetsov, he also received a doctorate for this dissertation. This was a fair assessment of this outstanding work.

### Additional information

**Fleroff's herbarium**

Fleroff's herbarium collections from Vladimir Governorate are now preserved in two herbaria, i.e. the Moscow University Herbarium (MW) and the Komarov Institute Herbarium (LE). The specimens collected in 1894–1901 document data from the original source (Fleroff 1902).

The MW Herbarium has been entirely digitised (Seregin 2018, Seregin 2021c) and, therefore, we can fully examine Fleroff’s collections. The LE Herbarium is still only on the way to digitisation; however, some specimens of rare plants collected by Fleroff were cited earlier by Seregin (2012).

The MW Herbarium holds 676 specimens collected by Fleroff in Vladimir Governorate in 1894–1896: nine specimens of fairly rare species are dated back to 1894 (Fig. 7) and many more to 1895 and 1896 (Fig. 8). These collections cover Aleksandrovsky, Pereslavsky and Yuryevsky Districts (north-west of the territory). In addition, 17 duplicate specimens from other districts are dated back to 1900 (Fig. 9).

The LE Herbarium contains later collections by Fleroff from Vladimir Governorate (1897–1907). Judging by the labels, the specimens for 1897, 1900 and 1901 were undoubtedly collected during the preparation of the original source (Fleroff 1902).

## Sampling methods

### Sampling description

Georeferencing of digitised species lists (see below) was carried out, based on the expert knowledge of the area, analysis of modern satellite images and old topographic maps. Fleroff's lists of routes, which he gave at the beginning of each chapter of the original source, were of great help for us. For each route, he gave a sequential list of localities (i.e. villages, stations, rivers, lakes etc.), which allows us to understand his transportations. The mean accuracy of records of the entire dataset is 2,447 m. For 2,460 records, the georeferencing accuracy is 1,000 m or less (28%), whereas for 6,070 records, it is 2,000 m or less (68%). That level of accuracy was unattainable for most herbarium collections of the late 19^th^ century.

### Step description

**1. List of species.** In the original source (Fleroff 1902), almost all species are given in Latin in the form of a two-column list for every plant community. These two-column lists include names of vascular plants without taxonomic authors (Fig. 5). Additionally, mosses and lichens are sometimes mentioned in the text. Textual description also contains a clear indication of the locality. Initially, we tried to digitise these lists through OCR, but the old font and the quality of the electronic version led to a number of errors in the name recognition. We retyped all Latin names de novo. The resulting list with page references included ca. 10,000 lines.

**2. Georeferences and their list.** Simultaneously, but independently from the first step, we made a spreadsheet of localities and communities studied and documented by Fleroff (1902) and their georeferences. The final spreadsheet included citations of the original Russian text for 494 individual communities studied by Fleroff.

We used two main sources for georeferencing: (1) modern satellite images and electronic maps of Yandex (https://yandex.ru/maps/) and a detailed digitised map by Mende of Vladimir Governorate, 1848–1850 (http://www.etomesto.ru/map-vladimir_mende/). From time to time, we have used other cartographic sources and textual descriptions of places from a wide variety of sources on the Internet. We georeferenced Fleroff's records to 367 centroids, because sometimes the author described several closely-situated communities within the same locality (for example, aquatic plants and coastal plants of the lake). The first map (Fig. 10a) shows a spatial distribution of the centroids across modern first-level administrative units, whereas the second map (Fig. 10b) gives an overview of the data density (i.e. a number of plant records per centroid).

Three places mentioned by Fleroff (1902) were not discovered: Gremyach Forest (near the City of Aleksandrov), Chertenovskoye peat bog (on the border of Aleksandrovsky and Pereslavsky Districts), Voloty locality on the Oka River (Melenkovsky District). One point was left unreferenced due to a typo in the original text (a village name mismatches a river name). Overall, the success rate of georeferencing was 99.2%. Using the internal geoservice of the Moscow Digital Herbarium, we linked the centroids to the modern administrative units of the Russian Federation, both the first-level units (oblasts) and the second-level units (districts, cities).

**3. Harmonisation of species lists and georeferences.** On this step, we merged and harmonised two spreadsheets, i.e. species lists by pages and a list of georeferenced localities. At this stage, the original source was always at hand. We identified and eliminated some accidental omissions and typos. Location descriptions were standardised. We excluded some Latin names mentioned without localities (for example, in conclusions or discussion).

**4. Excluding non-original data.** Fleroff (1902) actively used data from other published sources with direct and clear references to the primary sources. For instance, the most extensive borrowings were made by him for the eastern part of Melenkovsky District (now in Nizhny Novgorod Oblast) following the monograph by Bulatkin (1896). We completely excluded from the dataset all the data taken from external sources, i.e. Fleroff's non-original data. Fleroff (1902) gave the list of 27 references on pages VII–X of the original source.

**5. Adding records based upon the Russian vernacular names.** A remarkable feature of the book by Fleroff (1902) is the mentioning of some dominant plants in Russian without its duplication in Latin. Such records (318 records, 14 taxa) were additionally added to the dataset:

- birch - "береза" in Russian (*Betula*),
- heather - "вереск" in Russian (*Callunavulgaris* (L.) Hull),
- pedunculate oak - "дуб" in Russian (*Quercusrobur* L.),
- Norway spruce - "ель" in Russian (*Piceaabies* (L.) H. Karst.),
- willow - "ива" in Russian (*Salix*),
- common club-rush - "камыш" in Russian (*Schoenoplectuslacustris* (L.) Palla),
- common juniper - "можжевельник" in Russian (*Juniperuscommunis* L.),
- alder - "ольха" in Russian (*Alnus*),
- hazel - "орешник" in Russian (*Corylusavellana* L.),
- aspen - "осина" in Russian (*Populustremula* L.),
- black-poplar - "осокорь" in Russian (*Populusnigra* L.),
- Scots pine - "сосна" in Russian (*Pinussylvestris* L.),
- peat moss - "сфагны" in Russian (*Sphagnum*),
- common reed - "тростник" in Russian (*Phragmitesaustralis* (Cav.) Trin. ex Steud.).

**6. Cleaning list of species, synchronisation with a backbone.** We checked the list of re-typed names for errors of two kinds, i.e. typos in the original text and typos by the input operator. These cases have been standardised. The standardisation of orthography reduced the number of taxa entries from 766 to 678.

The orthographically-clean set of names was further synchronised with the nomenclature according to Seregin (2014b), which was recently published as a checklist dataset in GBIF (Seregin 2021d). This was the most crucial stage, since Fleroff's nomenclature, brilliant for 1902, is currently archaic and demanded a re-assessment of what Fleroff (1902) meant by this or that name. At this stage, two sources were actively involved: (1) nomenclatural paragraphs from the atlas (Seregin 2012) with complete assessment of the Fleroff's names and (2) Fleroff's herbarium. We automatically matched 397 names and cross-linked manually the remaining 282 names.

**7. DarwinCore format.** We transformed the final spreadsheet with 8,889 records into the DarwinCore format. It includes 20 variable fields, whereas an additional 28 constant fields were set directly in the IPT. After publication, the data cleaning procedure was based on the "Issues and flags" section on the dataset page (https://doi.org/10.15468/8qf7sh).

## Geographic coverage

### Description

A dataset covers Vladimir Governorate of the Russian Empire in the borders of 1901. Currently, records by Fleroff (1902) refer to 33 second-level administrative units of five oblasts (first-level administrative units) of the Russian Federation: Vladimir Oblast, Moscow Oblast, Yaroslavl Oblast, Ivanovo Oblast and Nizhny Novgorod Oblast (Table 1, Table 2).

The list of localities include some places completely transformed by human activity in the 20^th^ century. For instance, Berendeyevo Peat Bog has been drained and mined since 1918 (Fig. 11). Some localities studied by Fleroff (1902) were destroyed during the growth of urban residential areas of Karabanovo, Vladimir and Kovrov.

### Coordinates

55 and 57 Latitude; 37.5 and 43.5 Longitude.

## Taxonomic coverage

### Description

The checklist by Seregin (2021d) serves as a taxonomic backbone for this dataset, but it covers only tracheophytes. Additional names of bryophytes, lichens, green algae, hepatics and charophytes were given against the original text by Fleroff (1902), i.e. with no taxonomic authors. As a result, an occurrence dataset (Seregin and Basov 2021) includes 654 accepted scientific names.

The following species names by Fleroff (1902) cannot be implemented with certainty. They are listed in the occurrence dataset as generic names, based upon our current expert knowledge of the Vladimir Oblast flora:

- “*Agrostisalba*” was treated as *Agrostis* sp. (currently treated as *Agrostisstolonifera* and *Agrostisgigantea*)
- “*Agrostiscanina*” was treated as *Agrostis* sp. (currently treated as *Agrostiscanina* and *Agrostisvinealis*)
- “*Alchemillavulgaris*” was treated as *Alchemilla* sp. (currently treated as several dozens of microspecies)
- “*Arabisgerardi*” and “*Arabishirsuta*” were treated as *Arabis* sp. (showed that applied these names wrongly and partly mixed the species)
- “*Carexcontigua*” was treated as *Carex* sp. (records were made in peat bogs and clearly do not refer to *Carexspicata*)
- “*Euphrasiaofficinalis*” was treated as *Euphrasia* sp. (currently treated as several microspecies)
- “*Hieraciumauricula*” and “*Hieraciumpratense*” were treated as *Pilosella* sp. (Sennikov in Seregin (2012) insisted that interpretation of old *Pilosella* names should be based upon herbarium specimens)
- “*Isoeteslacustris*” was treated as *Isoetes* sp. (currently treated as *Isoeteslacustris* and *Isoetessetacea*)
- “*Koeleriacristata*” was treated as *Koeleria* sp. (Fleroff (1902) implemented this name to *Koeleriadelavignei* and partly to *Koeleriaglauca*)
- “*Lycopodiumcomplanatum*” was treated as *Diphasiastrum* sp. (currently treated as *Diphasiastrumcomplanatum*, *Diphasiastrum* x *zeilleri* and possibly *Diphasiastrumtristachyum*)
- “*Orchismaculata*” was treated as *Dactylorhiza* sp. (currently treated as *Dactylorhizafuchsii* and *Dactylorhizamaculata*)
- “*Ranunculusdivaricatus*” was treated as *Ranunculus* sp. (currently treated as *Ranunculuskauffmanii* and *Ranunculustrichophyllus*)
- “*Rumexmaximus*” was treated as *Rumex* sp. (Fleroff (1902) used this name once at page 35, but in the checklist, he did not mention his own record at all; probably, it refers to *R.aquaticus*)
- “*Salixstipularis*” was treated as *Salix* sp. (the only hybrid in *Salix* mentioned by Fleroff (1902); we left it unresolved in the absence of a voucher specimen)
- “*Tragopogonpratense*” was treated as *Tragopogon* sp. (currently treated as *Tragopogonpratensis* and *Tragopogonorientalis*)

### Taxa included

**Table:** 

Rank	Scientific Name	
phylum	Tracheophyta	
phylum	Bryophyta	
phylum	Marchantiophyta	
phylum	Chlorophyta	
phylum	Charophyta	
phylum	Ascomycota	

## Temporal coverage

**Data range:** 1894-1-01 – 1901-12-31.

### Notes

A book by Fleroff (1902) does not have precise indications of the years of the fieldwork. Since he spent his childhood and adolescence in the Kolpakovo Estate near the City of Aleksandrov, his observations of the Vladimir nature began in the school years. We used Fleroff's herbarium collections, his biography written by his son in 1981 (published in Seregin 2012) and occasional footnotes in the original source to establish the period of his field studies. Now we can say unequivocally that the book is based on Fleroff's field data collected in 1894–1901.

## Usage licence

### Usage licence

Creative Commons Public Domain Waiver (CC-Zero)

## Data resources

### Data package title

"Flora des Gouvernements Wladimir" (Fleroff, 1902): georeferenced records

### Resource link

https://www.gbif.org/dataset/d34156ac-83af-4c33-9686-cc71a1045826

### Alternative identifiers

https://doi.org/10.15468/8qf7sh, https://depo.msu.ru/ipt/archive.do?r=fleroff, https://depo.msu.ru/ipt/eml.do?r=fleroff

### Number of data sets

1

### Data set 1.

#### Data set name

"Flora des Gouvernements Wladimir" (Fleroff, 1902): georeferenced records

#### Data format

DarwinCore

#### Number of columns

48

#### Download URL

https://depo.msu.ru/ipt/archive.do?r=fleroff

#### Description

8,889 georeferenced records of 654 taxa from the first part of "Flora des Gouvernements Wladimir" (Fleroff 1902), which include species lists by localities studied by the author in 1894-1901. The nomenclature is given against Seregin, A.P. 2014. Flora of Vladimir Oblast, Russia: Grid data analysis. Moscow, KMK Scientific Press. 441 p. ISBN 978-5-9905832-9-0 (http://dx.doi.org/10.13140/2.1.1148.2407).

**Data set 1.** 

Column label	Column description
occurrenceID	An identifier for the Occurrence (as opposed to a particular digital record of the occurrence). A variable constructed from a combination of two identifiers in the record that will most closely make the occurrenceID globally unique (datasetID + ID of a record within the dataset). For example, "'urn:lsid:biocol.org:col:15550:11:5030".
dcterms:type	The nature or genre of the resource. A constant ("Dataset").
dcterms:modified	The most recent date-time on which the resource was changed. A constant ("2021-09-04").
dcterms:language	A language of the resource. A constant ("en | ru", i.e. English and Russian)
dcterms:license	A legal document giving official permission to do something with the resource. A constant ("http://creativecommons.org/licenses/by/4.0/legalcode").
dcterms:rightsHolder	A person or organisation owning or managing rights over the resource. A constant ("Moscow State University").
dcterms:accessRights	Information about who can access the resource or an indication of its security status. A constant ("Use under CC BY 4.0").
institutionID	An identifier for the institution having custody of the object(s) or information referred to in the record. A constant ("http://grbio.org/institution/moscow-stateuniversity" for the Moscow State University).
collectionID	An identifier for the collection or dataset from which the record was derived. A constant ("urn:lsid:biocol.org:col:15550" for the Moscow University Herbarium).
datasetID	An identifier for the set of data. May be a global unique identifier or an identifier specific to a collection or institution. A constant ("urn:lsid:biocol.org:col:15550:11").
institutionCode	The name (or acronym) in use by the institution having custody of the object(s) or information referred to in the record. A constant ("Moscow State University").
datasetName	The name identifying the dataset from which the record was derived. A constant ("Flora des Gouvernements Wladimir" (Fleroff, 1902): georeferenced records).
ownerInstitutionCode	The name (or acronym) in use by the institution having ownership of the object(s) or information referred to in the record. A constant ("Moscow State University").
basisOfRecord	The specific nature of the data record - a subtype of the dcterms:type. A constant ("HumanObservation").
catalogNumber	An identifier (preferably unique) for the record within the dataset or collection. A variable. For example, "Flerov:5030".
recordedBy	A list (concatenated and separated) of names of people, groups or organisations responsible for recording the original occurrence. A variable. For example, "Alexander F. Fleroff".
occurrenceStatus	A statement about the presence or absence of a taxon at a location. A constant ("present").
associatedReferences	A list (concatenated and separated) of identifiers (publication, bibliographic reference, global unique identifier, URI) of literature associated with the Occurrence. A variable with a page reference. For example, "Fleroff (1902), p. 182 [Fleroff A. (1902). Flora des Gouvernements Wladimir. I. Pflanzengeographische Beschreibung des Gouvernements Wladimir. Moskva. 338 p.]".
eventDate	The date or interval during which an event occurred. For occurrences, this is the date when the event was recorded. A constant ("1894/1901").
higherGeography	A list (concatenated and separated) of geographic names less specific than the information captured in the locality term. A variable. For example, "Europe | Russian Federation | Vladimir Oblast | Petushinskii raion".
continent	The name of the continent in which the location occurs. A constant ("Europe").
country	The name of the country or major administrative unit in which the location occurs. A constant ("Russian Federation").
countryCode	The standard code for the country in which the location occurs. A constant ("RU").
stateProvince	The name of the next smaller administrative region than country (state, province, canton, department, region etc.) in which the location occurs. A variable. For example, "Vladimir Oblast".
county	The full, unabbreviated name of the next smaller administrative region than stateProvince (county, shire, department, etc.) in which the Location occurs. A variable. For example, "Petushinskii raion".
verbatimLocality	The original textual description of the place. A variable. For example, "озеро Верхнее по р. Ушма, берега".
locationRemarks	Comments or notes about the Location. A constant ("original description in Russian by Fleroff (1902)")
decimalLatitude	The geographic latitude (in decimal degrees, using the spatial reference system given in geodeticDatum) of the geographic centre of a location. A variable.
decimalLongitude	The geographic longitude (in decimal degrees, using the spatial reference system given in geodeticDatum) of the geographic centre of a location. A variable.
geodeticDatum	The ellipsoid, geodetic datum or spatial reference system (SRS) upon which the geographic coordinates given in decimalLatitude and decimalLongitude are based. A constant ("WGS84").
coordinateUncertaintyInMeters	The horizontal distance (in metres) from the given decimalLatitude and decimalLongitude describing the smallest circle containing the whole of the location. A variable.
coordinatePrecision	A decimal representation of the precision of the coordinates given in the decimalLatitude and decimalLongitude. A constant ("0.0001").
georeferencedBy	A list (concatenated and separated) of names of people, groups or organisations who determined the georeference (spatial representation) of the location. A constant ("Alexey P. Seregin").
georeferencedDate	The date on which the Location was georeferenced. A constant ("2021-08").
georeferenceSources	A list (concatenated and separated) of maps, gazetteers or other resources used to georeference the Location, described specifically enough to allow anyone in the future to use the same resources. A constant ("https://yandex.ru/maps/ | http://www.etomesto.ru/map-vladimir_mende/").
georeferenceRemarks	Notes or comments about the spatial description determination, explaining assumptions made in addition or opposition to the those formalised in the method. A variable. For example, "centroid position: у Бельских двориков".
identifiedBy	A list (concatenated and separated) of names of people, groups or organisations who assigned the Taxon to the subject. A constant ("Alexander F. Fleroff").
dateIdentified	The date on which the subject was identified as representing the Taxon. A constant ("1894/1901").
taxonID	An identifier for the set of taxon information (data associated with the Taxon class). May be a global unique identifier or an identifier specific to the dataset. A variable. For example, "VLA0034" as a reference for *Pinussylvestris* L. in https://doi.org/10.15468/7zk2y5.
nameAccordingToID	An identifier for the source in which the specific taxon concept circumscription is defined or implied. See nameAccordingTo. A variable. For example, doi: 10.15468/7zk2y5.
scientificName	The full scientific name, with authorship and date information, if known. A variable (for example, "Scirpus sylvaticus L.").
nameAccordingTo	For taxa that result from identifications, a reference to the keys, monographs, experts and other sources should be given. A variable. Two options: (1) "Seregin A (2021). Flora of Vladimir Oblast (Seregin, 2014): accepted names. Lomonosov Moscow State University. Checklist dataset https://doi.org/10.15468/7zk2y5 accessed via GBIF.org on 2021-09-04"; (2) "Fleroff A. (1902). Flora des Gouvernements Wladimir. I. Pflanzengeographische Beschreibung des Gouvernements Wladimir. Moskva. 338 p."
phylum	The full scientific name of the phylum or division in which the taxon is classified. A variable. For example, "Tracheophyta".
taxonRank	The taxonomic rank of the most specific name in the scientificName. A variable. (four options: "species", "variety", "genus", "speciesAggregate").
vernacularName	A common or vernacular name. A variable. For example, "сфагны".
nomenclaturalCode	The nomenclatural code (or codes in the case of an ambiregnal name) under which the scientificName is constructed. A constant ("International Code of Nomenclature for algae, fungi and plants").
taxonomicStatus	The status of the use of the scientificName as a label for a taxon. A constant ("accepted").
taxonRemarks	Comments or notes about the taxon or name. A variable. For example, "тростник in Fleroff (1902)".

## Figures and Tables

**Figure 4a.**
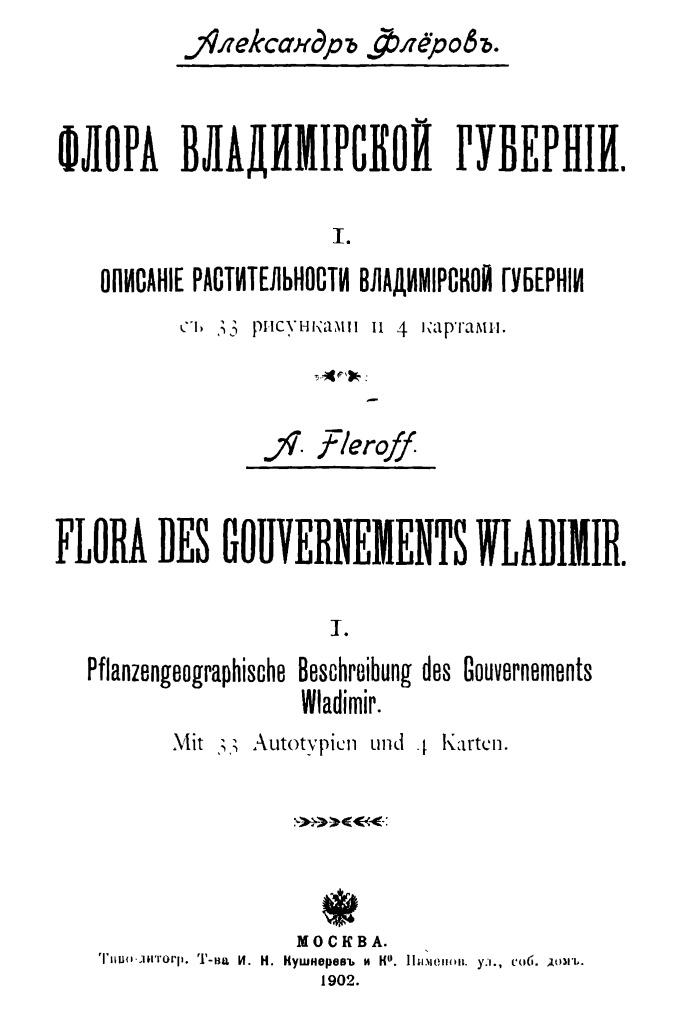
title page of "Flora des Gouvernements Wladimir. I. Pflanzengeographische Beschreibung des Gouvernements Wladimir" (in Russian, with enlarged German abstract), the first part.

**Figure 4b.**
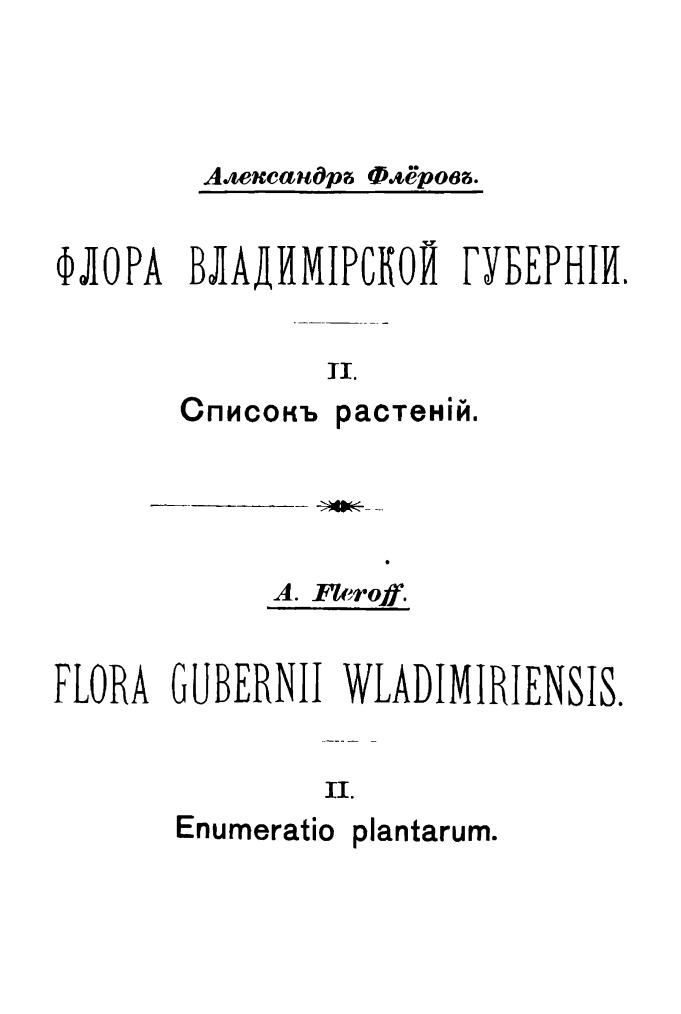
title page of "Flora Gubernii Wladimiriensis. II. Enumeratio plantarum" (in Latin), the second part.

**Figure 5a.**
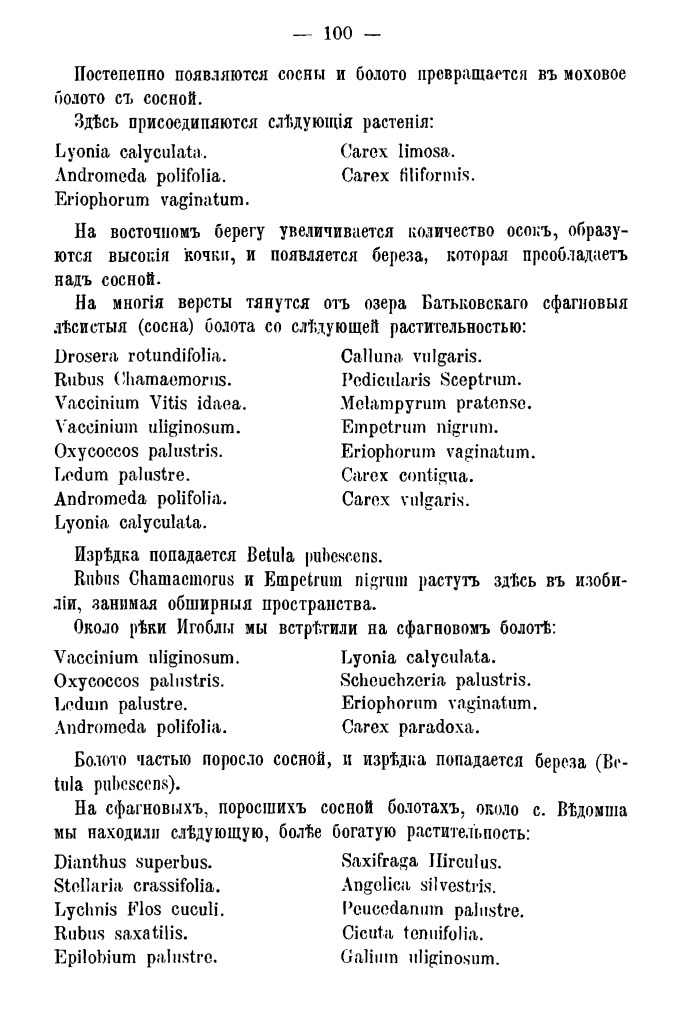
species lists for Lake Batkovskoye (including noteworthy records of *Rubuschamaemorus* L. and *Empetrumnigrum* L.) and vicinity of Vedomsha (with a record of *Saxifragahirculus* L.), page 100

**Figure 5b.**
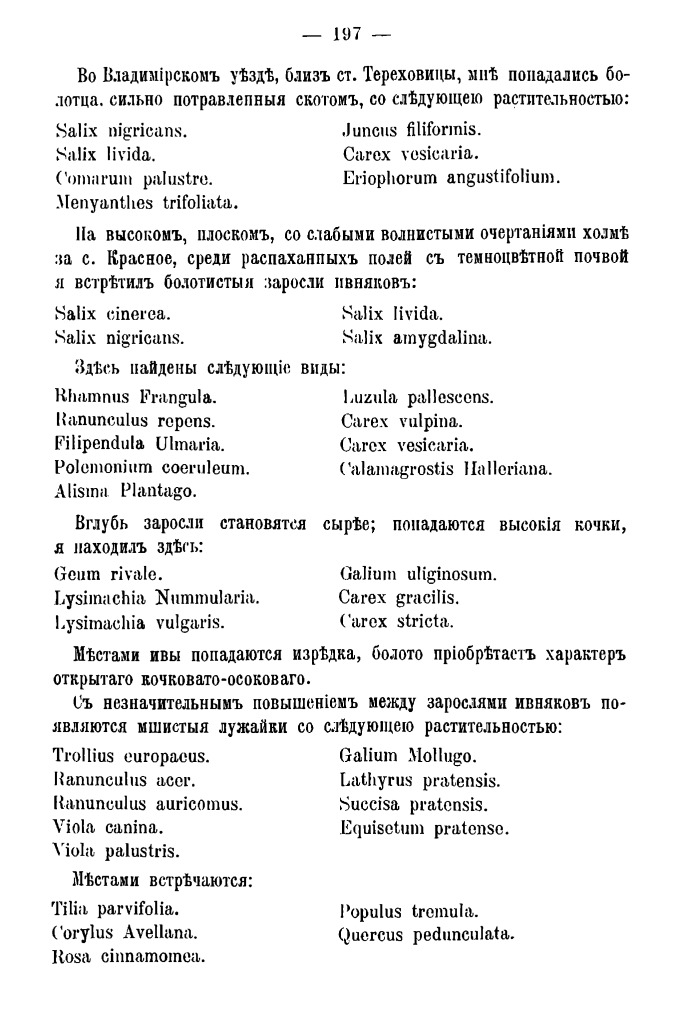
species lists for the vicinity of Terekhovitsy railway station and Krasnoye (plant communitites from the latter locality are completely lost due to the growth of residential areas of the City of Vladimir in the 20^th^ century), page 197.

**Figure 6a.**
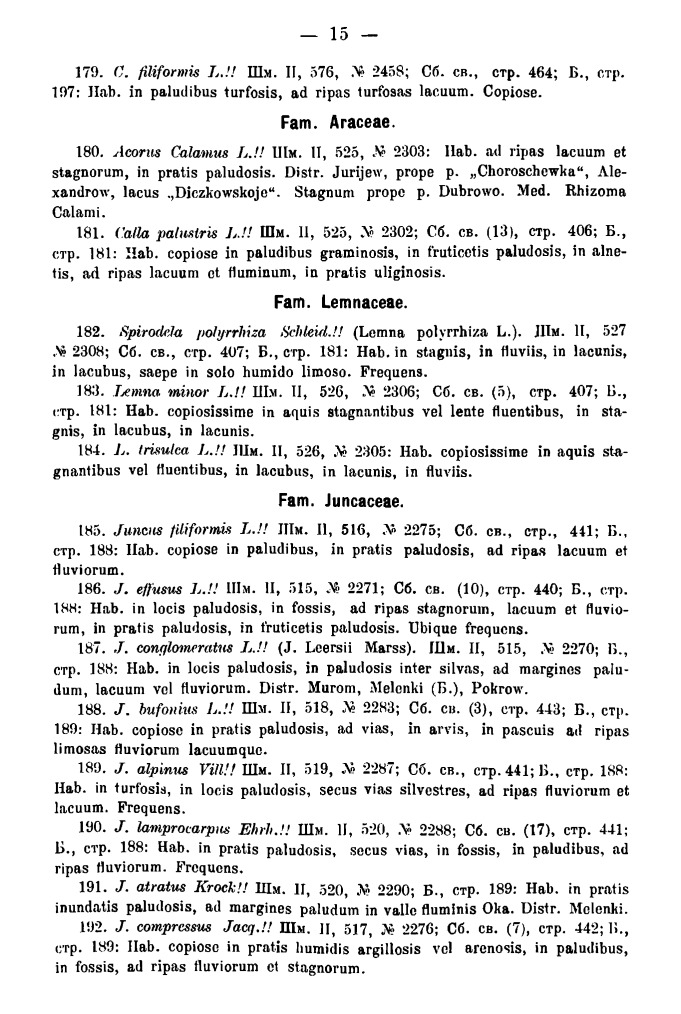
Araceae to Juncaceae, page 15.

**Figure 6b.**
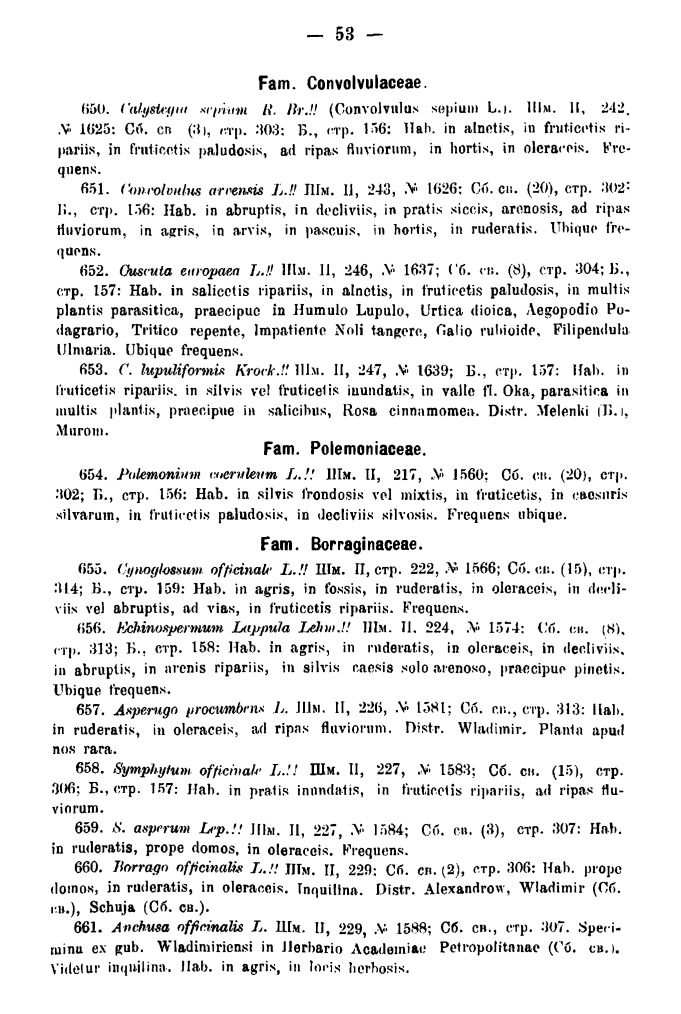
Convolvulaceae to Boraginaceae, page 53.

**Figure 7a.**
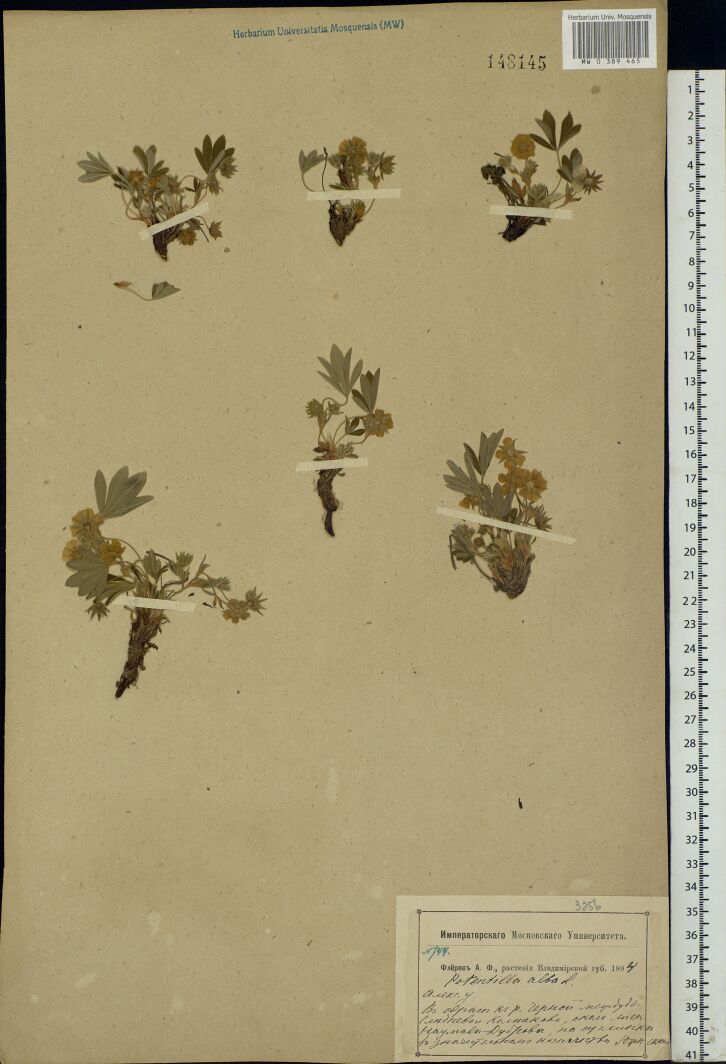
This herbarium specimen was collected by Fleroff in his time as a student of the Moscow University. *Potentillaalba* L. is one of the most noteworthy records made by him.

**Figure 7b.**
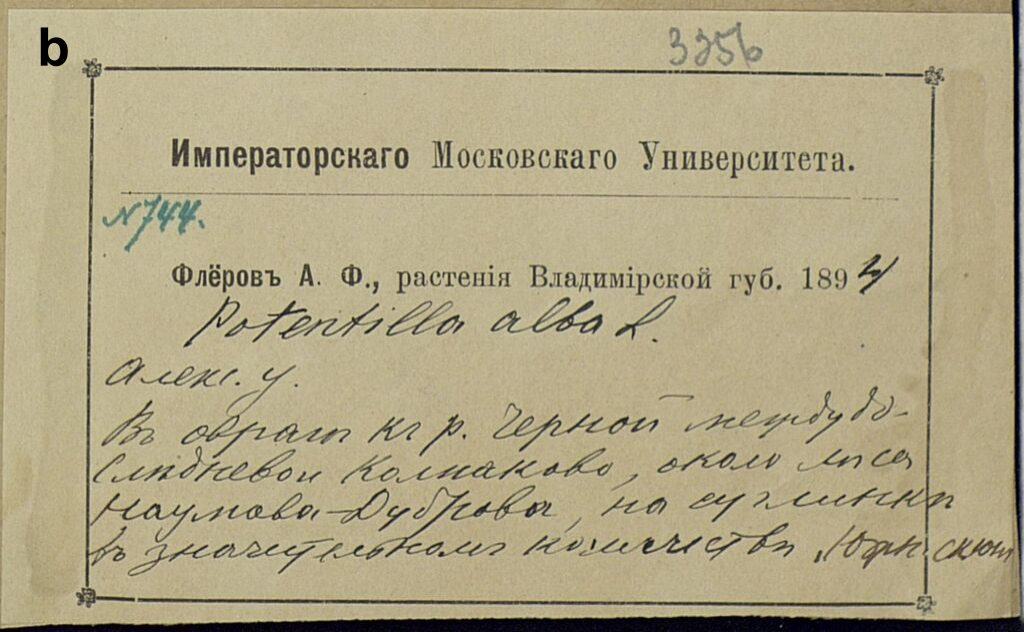
In 1894, herbarium collections by Fleroff were perfectly documented. For instance, "Fleroff A.F., plants of Vladimir Governorate. 1894. *Potentillaalba* L. Aleksandrovsky District. In the ravine to the River Chernaya between Slednevo and Kolpakovo, near the Naumova Dubrova Forest, on loam, in a significant number, southern slope. № 744".

**Figure 8a.**
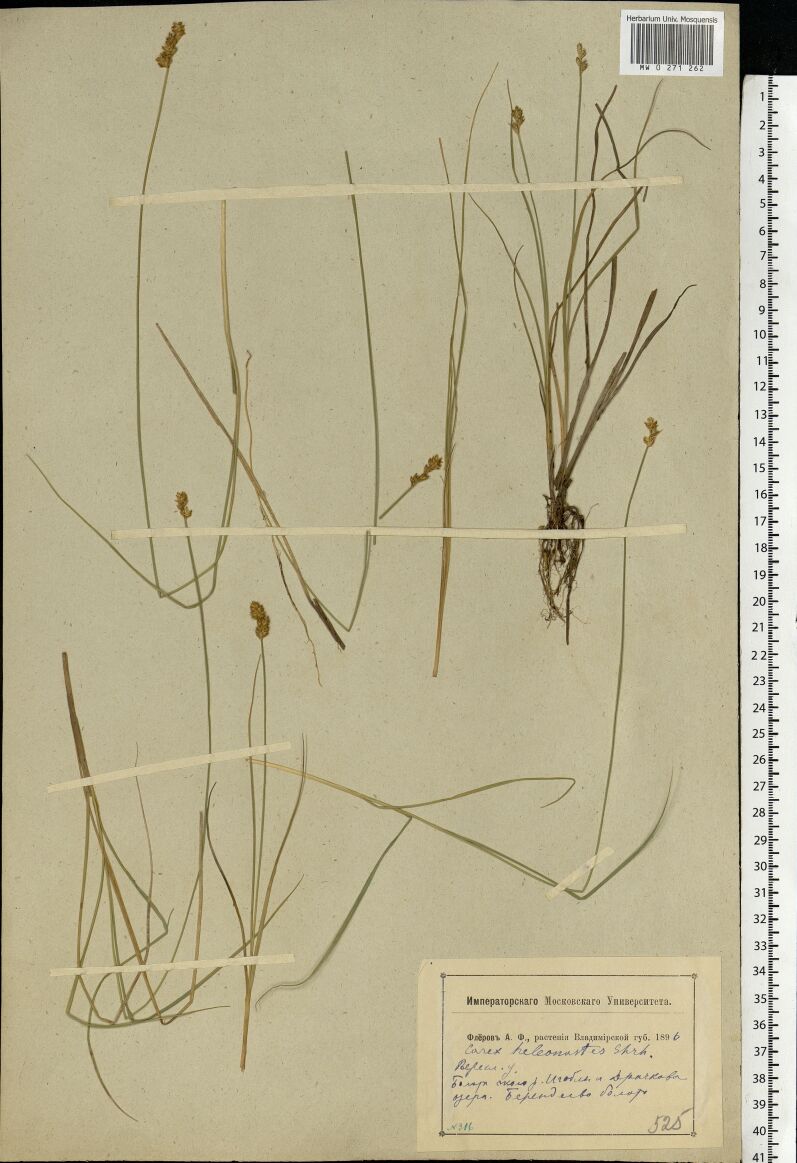
This herbarium specimen was collected by Fleroff two years later, in 1896. The southernmost localities of *Carexheleonastes* Ehrh. ex L.f. in European Russia are also amongst the most noteworthy records made by him.

**Figure 8b.**
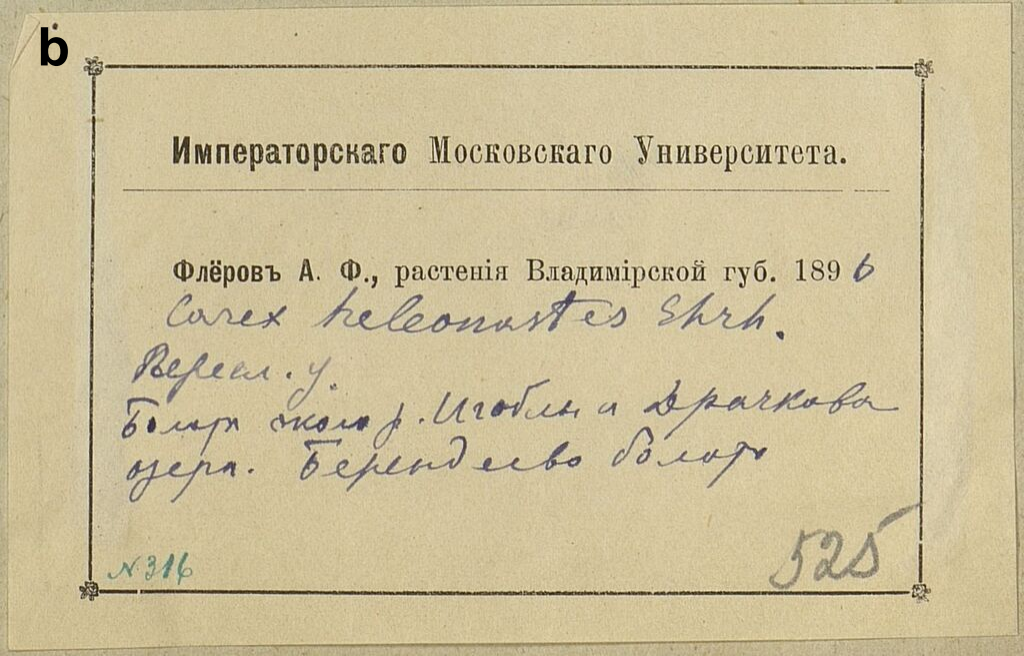
In 1896, herbarium labels by Fleroff became less specific even for rarities. For instance, "Imperial Moscow University. Fleroff A.F., plants of Vladimir Governorate. 1896. *Carexheleonastes* Ehrh. Pereslavsky District. Mires near the Igobla River and Lake Drachkovo. Berendeyevo peat bog. № 316". Three localities are listed in a single label with no indication where the plants were exactly collected.

**Figure 9a.**
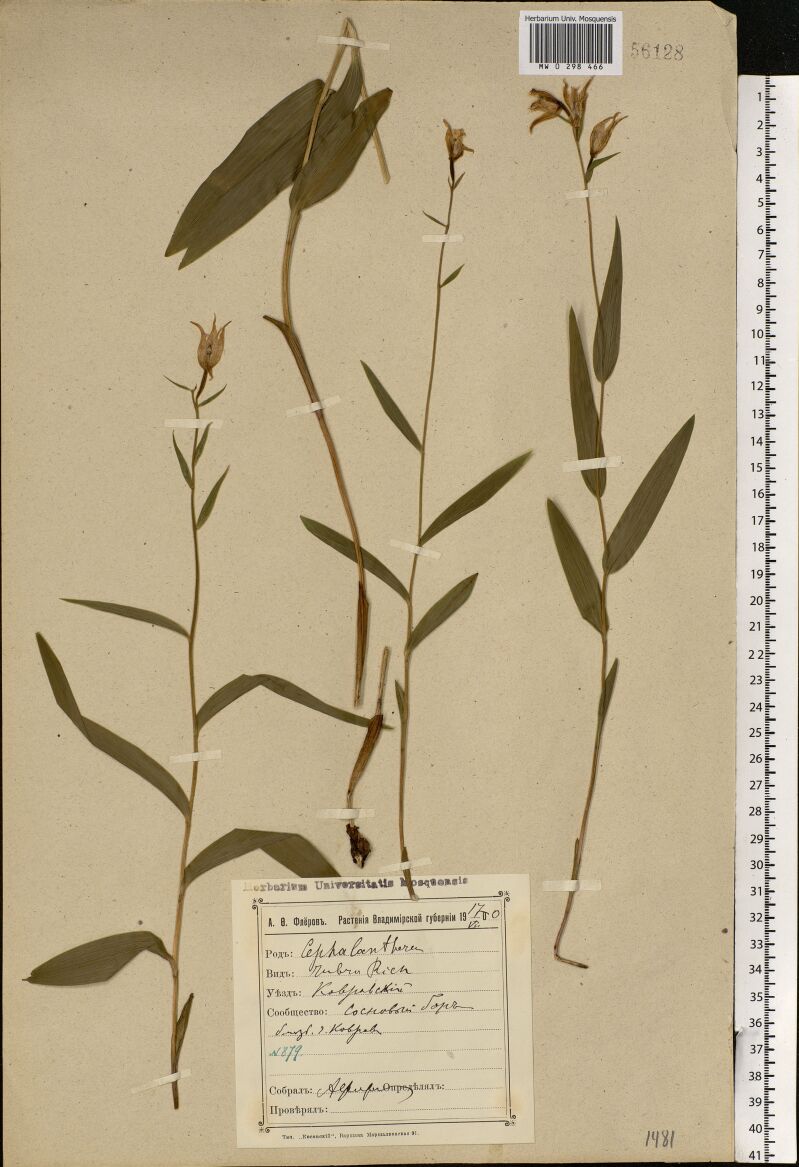
This herbarium specimen was collected by Fleroff during his extensive trips across Vladimir Governorate in 1900 undertaken for his Master's Thesis "Flora des Gouvernements Wladimir". *Cephalantherarubra* (L.) Rich. was discovered in pinewoods on calcareous ground near Kovrov.

**Figure 9b.**
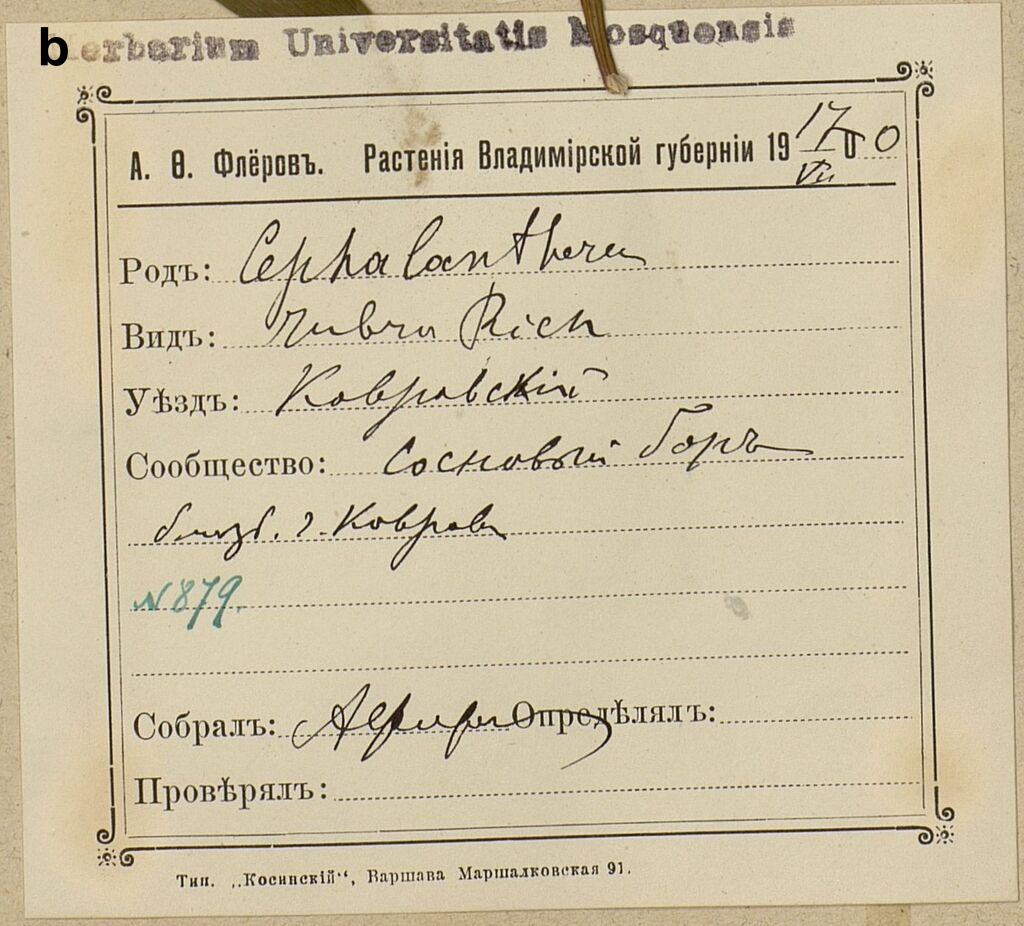
In 1900, herbarium labels by Fleroff became more specific and included a date of collection. For instance, "A.F. Fleroff. Plants of Vladimir Governorate. 17.VII.1900. *Cephalantherarubra* Rich. District: Kovrovsky. Community: pine forest near the City of Kovrov. № 879. Collected by A. Fleroff". There were no affiliation with the Imperial Moscow University on his labels at that time. A blank label was printed in Warsaw.

**Figure 10a.**
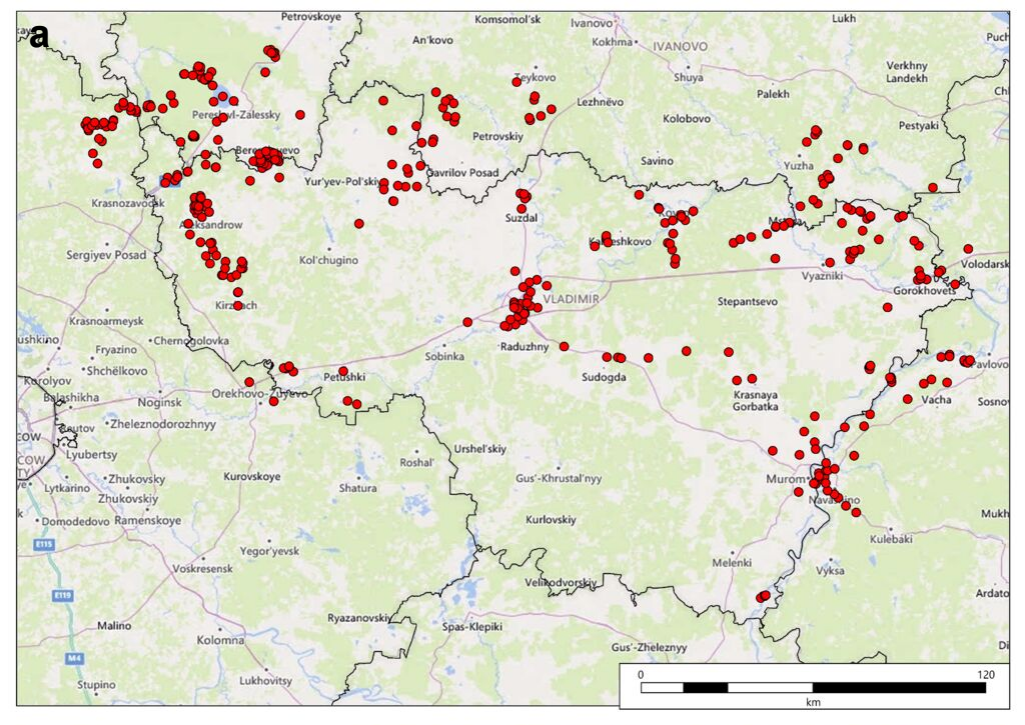
Spatial distribution of centroids.

**Figure 10b.**
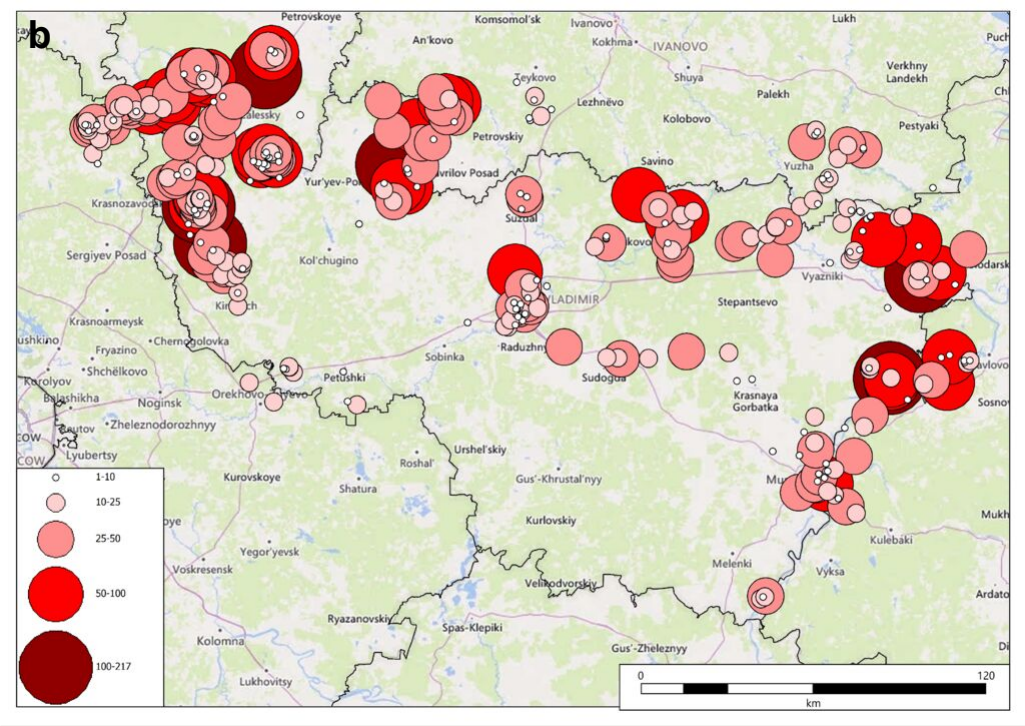
Spatial distribution of weighted data (circles show the number of records per centroid).

**Figure 11a.**
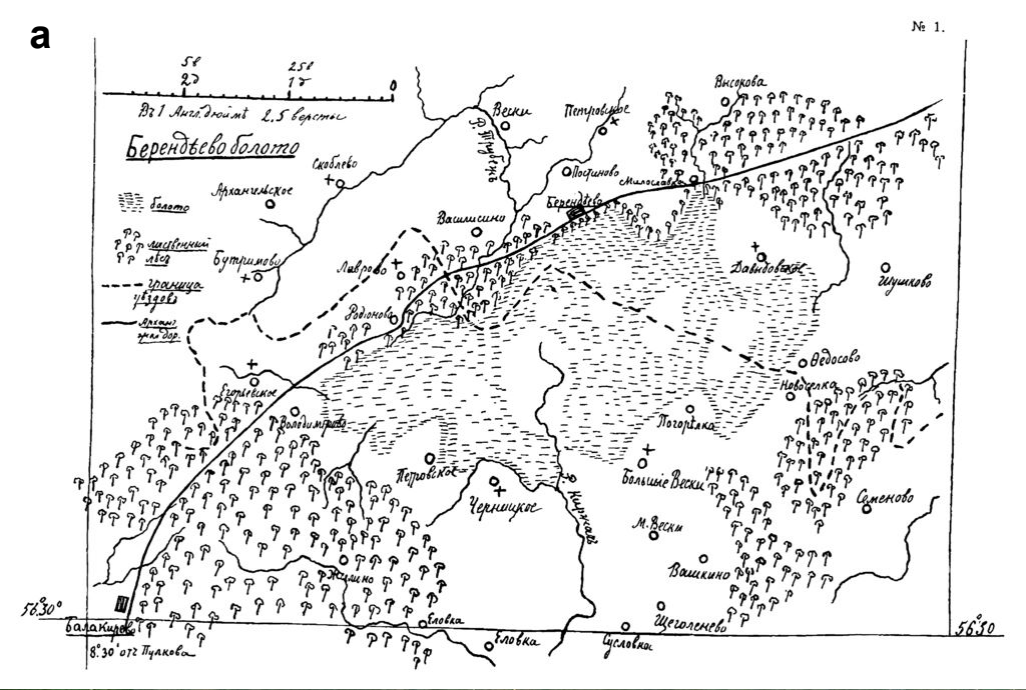
Sketch map from the original source of the virgin peat bog.

**Figure 11b.**
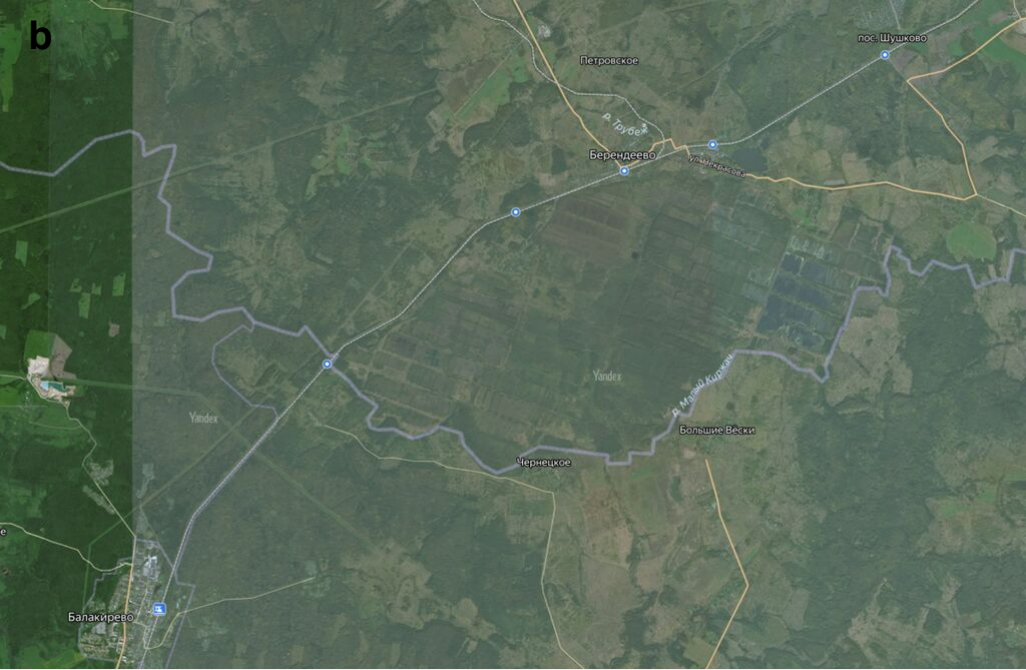
Modern satellite image (available at https://yandex.ru/maps/) showing flooded peat milling fields of the 20^th^ century.

**Table 1.** General overview of digitised data from Fleroff (1902) against modern first-level administrative units (oblasts) of the Russian Federation.

**Modern region**	**Number of centroids**	**Number of species**	**Number of records**
Vladimir Oblast	195	534	4,611
Yaroslavl Oblast	66	409	2,013
Nizhny Novgorod Oblast	37	307	942
Ivanovo Oblast	36	273	667
Moscow Oblast	32	203	656
Total	367	654	8,889

**Table 2.** General overview of digitised data from Fleroff (1902) against modern second-level administrative/municipal units (districts and cities) of the Russian Federation.

**Modern district**	**Modern region**	**Number of centroids**	**Number of species**	**Number of records**
Pereslavsky District	Yaroslavl Oblast	66	409	2,013
Aleksandrovsky District	Vladimir Oblast	40	317	1,318
Sergievo-Posadsky District	Moscow Oblast	29	199	599
Yuryev-Polsky District	Vladimir Oblast	14	257	586
Vachsky District	Nizhny Novgorod Oblast	13	249	523
Suzdalsky District	Vladimir Oblast	28	200	482
Vyaznikovsky District	Vladimir Oblast	26	187	399
Gorokhovetsky District	Vladimir Oblast	14	190	375
Gavrilovo-Posadsky District	Ivanovo Oblast	11	202	368
Navashinsky District	Nizhny Novgorod Oblast	16	144	283
Kovrovsky District	Vladimir Oblast	11	134	258
Kirzhachsky District	Vladimir Oblast	13	132	258
Yuzhsky District	Ivanovo Oblast	18	137	244
City of Kovrov	Vladimir Oblast	5	113	193
Kameshkovsky District	Vladimir Oblast	6	102	139
Sudogodsky District	Vladimir Oblast	6	76	123
Muromsky District	Vladimir Oblast	7	83	108
Melenkovsky District	Vladimir Oblast	4	76	94
Petushinsky District	Vladimir Oblast	8	63	85
City of Vladimir	Vladimir Oblast	4	55	65
Volodarsky District	Nizhny Novgorod Oblast	2	57	64
Teykovsky District	Ivanovo Oblast	7	41	55
Pavlovsky District	Nizhny Novgorod Oblast	5	44	53
City of Murom	Vladimir Oblast	1	40	45
Town of Aleksandrov	Vladimir Oblast	1	37	42
Orekhovo-Zuyevsky District	Moscow Oblast	2	30	39
Selivanovsky District	Vladimir Oblast	3	22	24
Kulebaksky District	Nizhny Novgorod Oblast	1	17	19
Taldomsky District	Moscow Oblast	1	15	18
Town of Suzdal	Vladimir Oblast	1	7	7
Sobinsky District	Vladimir Oblast	1	1	1
Town of Vyazniki	Vladimir Oblast	1	1	1
Kolchuginsky District	Vladimir Oblast	1	1	1
